# Cross-Voting SVM Method for Multiple Vehicle Classification in Wireless Sensor Networks

**DOI:** 10.3390/s18093108

**Published:** 2018-09-14

**Authors:** Heng Zhang, Zhongming Pan

**Affiliations:** College of Artificial Intelligence, National University of Defense Technology, Changsha 410073, China; chungmingpan@nudt.edu.cn

**Keywords:** multi-class classification, cross-voting SVM method, vehicle classification, wireless sensor networks (WSNs)

## Abstract

A novel multi-class classification method named the voting-cross support vector machine (SVM) method was proposed in this study, for classifying vehicle targets in wireless sensor networks. The advantages and disadvantages of available methods were summarized, after a comparative analysis of commonly used multi-objective classification algorithms. To improve the classification accuracy of multi-class classification and ensure the low complexity of the algorithm for engineering implementation on wireless sensor network (WSN) nodes, a framework was proposed for cross-matching and voting on the category to which the vehicle belongs after combining the advantages of the directed acyclic graph SVM (DAGSVM) method and binary-tree SVM method. The SVM classifier was selected as the basis two-class classifier in the framework, after comparing the classification performance of several commonly used methods. We utilized datasets acquired from a real-world experiment to validate the proposed method. The calculated results demonstrated that the cross-voting SVM method could effectively increase the classification accuracy for the classification of multiple vehicle targets, with a limited increase in the algorithm complexity. The application of the cross-voting SVM method effectively improved the target classification accuracy (by approximately 7%), compared with the DAGSVM method and the binary-tree SVM method, whereas time consumption decreased by approximately 70% compared to the DAGSVM method.

## 1. Introduction

Wireless sensor networks (WSNs) consist of nodes capable of sensing, signal processing, and communicating. In addition to object detection, the inherent property of control and activation in WSNs is also of interest [1]. One of the most significant applications of WSNs is in the classification of moving vehicles in designated areas, which is an important signal processing task and has found widespread civilian applications, such as in intelligent transportation systems [2] and real-time traffic surveillance [3].

Multi-class classification algorithm is developed based on a two-class classification algorithm. The commonly used methods for two-class classification are the decision tree (DT) method [4], naïve Bayesian (NB) method [5], ensemble method [6] and support vector machine (SVM) method [7]. The available multi-class classification algorithms for classifying multiple types of vehicles detected in the WSN system can be divided into two categories. The first category of multi-class classification method improves on the principle of two-class classification algorithm. Another multi-target classification category utilizes multiple two-class classifiers to classify multiple targets. The first approach exhibits high computational complexity, whereas the second approach requires pre-training of multiple two-class classifiers. 

There are numerous new research studies on the first type of method, including the MC-SVMA model [8], the K-class support vector classification-regression (K-SVCR) method [9], the learning vector quantization (LVQ) method [10], the improving prototype selection k-nearest neighbor (PS-KNN) method [11], Bayesian network-based chain classifiers [12], the BPSO-AdaBoost-KNN ensemble learning algorithm [13], and the hyper-sphere multi-class SVM (HSMC-SVM) method [14]. Among these, the algorithm that improved over the SVM principle for classifying multiple targets is the most widely applied. In addition to the MC-SVMA method and HSMC-SVM method mentioned above, the second-order cone programming support vector machines (SCOPSVM) method [15] and the least squares twin K-class support vector classification (LST-KSVC) method [16], are two other multi-class classification methods based on the SVM method.

The multi-class classification algorithm based on the two-class SVM classifier is the most widely used in the second category of multi-class classification methods, which includes the DAGSVM method [17], the one-against-all SVM (OAA-SVM) method [18], the binary-tree SVM [19] method, the hierarchical binary-tree multi-class SVM (BTMSVM) method [20], and decision tree DAGs [21]. 

Of the algorithms mentioned above, a few can classify vehicle type in WSNs by using the acoustic signal, seismic signal, or magnetic signal of the target acquired by a single sensor node in WSNs. The first category of multi-class classification methods is based on the improvement of the two-class classifier, and substantially increases the computational complexity of the algorithm. The limited storage space and computing capacity of the WSN node, hinder the implementation of the complex of classification algorithm on the node’s chip, and therefore the first type of algorithm, which is marginally more complicated, and is unable to apply for classifying vehicle types. For example, even when running on a computer, the HSMC-SVM algorithm takes 5–7 s to classify 1397 datapoints using trained classifiers [14], whilst LST-KSVC takes about 73 s to classify 5000 datapoints [16]. For WSN nodes, the complexity of the second type of methods are acceptable. For example, in this paper, the time taken by DAGSVM to classify averaging about 1060 16-dimensional vectors of target signals is 1.12 s, and the time consumed by the binary-tree to classify averaging about 1060 16-dimensional vectors is 0.28 s. The new algorithm aims to improve the accuracy of target classification, whilst rendering the increment of the computational complexity acceptable.

The rest of this paper is organized as follows. After introducing the feature extraction method, wavelet coefficient energy rate (WCER) method [22], and the dataset utilized for validating the proposed method, Section 2 compares the performances of commonly-used two-class classifiers. Section 3 compares the two available precursor algorithms of multi-class classification algorithms. Based on this comparison, Section 4 introduces the proposed voting-cross algorithm for vehicle classification in WSNs. The performance of the method is then discussed in Section 5, and Section 6 concludes the paper.

## 2. Comparison of Two-Class Classifiers

Prior to exploring the multi-target classification algorithm for WSN systems, we compare the two-class classification algorithms, which is the basis of multi-classification algorithms. Moreover, the wavelet coefficient energy ratio (WCER) feature extraction method was utilized to validate the classification algorithms, and the source datasets of targets were acquired from the third SensIT situational experiment [23].

### 2.1. Datasets and Feature Extraction Method

The datasets utilized in this study were gathered from a real-world experiment, the third SensIT situational experiment. In the experiment, 75 sensor nodes with acoustic, seismic, and infrared sensing capability were located at the Marine Corps Air Ground Combat Center. Testing runs were performed by driving different types of vehicles across the testing field. Four target vehicle-classes were used: Assault amphibian vehicle (AAV), Main battle tank, High-mobility multipurpose wheeled vehicle, and the Dragon wagon (DW). The acoustic and seismic data of each run were recorded, at a signal sampling rate of 4096 Hz. There were three paths in the experimental area for the vehicles to pass, and a testing run was accomplished by driving the vehicle on the path. A series of numbers was utilized to indicate these runs, e.g., DW3, DW4, and DW5. The acoustic and seismic data of each run were recorded by the sensor nodes deployed at the side of the road. The details of the experiment are described in literature in Reference [23].

**Figure 1:**
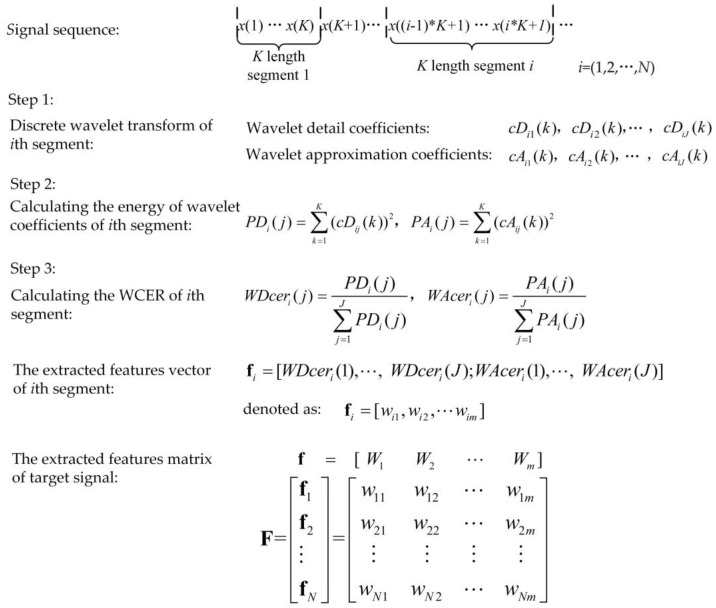
Process of feature extraction using wavelet coefficient energy ratio (WCER).

The WCER method, based on wavelet decomposition, was utilized for extracting the feature of the target signal after a specified length of signal sequence was acquired. Assuming that the feature of the target signal sequence was extracted at every K points, the process of the feature extraction method based on WCER is shown in Figure 1.

Figure 1 shows the process of the feature extraction method based on WCER, where J is the desired wavelet decomposition depth, *K* is the length of each segment of the signal, cDj (*k*) (*k* = 1, 2, …, *K*) are the wavelet detail coefficients, and cAj (*k*) is the approximation coefficient of the *j*th level. Then, we utilize the feature extraction method based on WCER to process the real-world, acquired signals of vehicles in WSNs. 

In this study, the length of the segment of the target signal sequence was set to 1024, and the desired wavelet decomposition depth J was set to 8. A nearly orthogonal design of the biorthogonal filter bank was utilized as the orthogonal filter bank to decompose the signal sequence. The wavelet decomposition process of the signal sequence is described in detail in our previous work [22].

### 2.2. Comparison of Performance of Different Two-Class Classifiers

The commonly used two-class classification methods are the k-nearest neighbor (KNN) method [24], the DT method, the NB method, the adaptive boosting (AdaBoost) [25] method, and the SVM method. We used the seismic feature data of AAV3 and AAV4 to train the classifiers and classify the seismic feature data of the other 15 runs (AAV5–AAV11 and DW5–DW12). Then, the average classification accuracy and time required were recorded to compare the performance of these methods, in terms of vehicle classification. 

The classification accuracy is denoted as *Ac*. We evaluated the accuracy as follows:

(1) $$Ac=\frac{\#\mathrm{correctly}\;\mathrm{classified}\;\mathrm{data}}{\#\mathrm{total}\;\mathrm{testing}\;\mathrm{data}}\times 100\%$$

The classification accuracy of the *i*th dataset is denoted as *Ac* (*i*) (*i* = 1, 2, …, *N*), and the average classification accuracy, *Aca*, is

(2) $$Aca=\frac{1}{N}\overset{N}{\underset{i=1}{\sum }}Ac\left(i\right)$$

where *N* is the number of datasets (acquired from different targets) that have been classified.

The classification results of the classification methods in Table 1, reveals that the classification accuracy of KNN is the highest in the two-class classification; however, its time consumption is excessive. The time consumptions of NB and DT for target classification were the lowest; however, the classification accuracy was low. Compared to the other classifiers, the SVM classifiers and AdaBoost exhibited reasonable performance; the classification was more accurate and required less time for subsequent classifications. The SVM classifier was more suitable for classifying the detected vehicle signals in the WSNs.

In practical applications, multiple types of targets would appear in the WSN system. Therefore, it is necessary to study multi-target classification method based on two-class SVM classifiers.

## 3. Comparison of Multi-Classes SVM Classifier

As summarized in Section 1, the available SVM algorithms for multi-target classification are divided into two categories. The first category improves on the principle of two-class classifier classification, for application to multi-target classification. Another multi-target classification category utilized multiple two-class classifiers to classify multiple targets. 

### 3.1. M-RLP SVM Method

**Figure 2:**
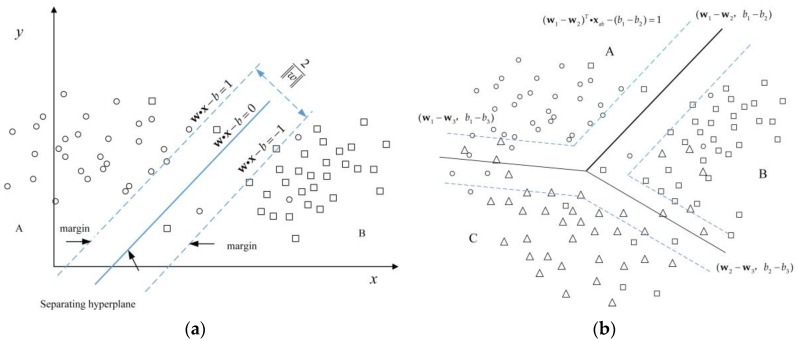
Two-class support vector machine (SVM) classifier and three-class SVM classifier: (**a**) two-class datasets separated by hyperplane; (**b**) three-class datasets separated by piecewise linear function.

As shown in Figure 2a, under ideal conditions, the two-class SVM method is aimed at searching for the optimal parameter that provides the largest distance between the margins of the datasets of two targets, according to the sample dataset. This is described mathematically as follows:

(3) $$min(\frac{1}{2}w^{T}w)$$

subject to

(4) $$y_{i}\left(w^{T}x_{i}+c\right)\geq 1,\;i=1,\cdots ,N_{t}$$

where **w** is the parameter of the classification hyperplane, *x_i_* is the classification sample point on the boundary, and *y_i_* is the label, and its value is either −1 or +1. *T* is the matrix transpose symbol and the constant *c* is the distance from the origin to the hyperplane.

Considering the mixed situation between the two types of sample datasets, the two-class SVM for dataset classification is aimed at solving the following optimization problem in practical applications:

(5) $$min(\frac{1}{2}w^{T}w+R\overset{N_{t}}{\underset{i=1}{\sum }}\varepsilon_{i})$$

under the following constraints:

(6) $$1-\varepsilon_{i}-y_{i}\left(w^{T}x_{i}+c\right)\leq 0,\;\varepsilon_{i}\geq 0,i=1,\cdots ,N_{t}$$

where *R* > 0 is the penalty factor, indicating the importance attached to the outliers, and *ε_i_* is the slack variable introduced when the *i*th sample data is linearly inseparable.

Figure 2b shows a multi-class SVM (MC-SVM) approach named Multi-class Robust Linear Programming (M-RLP) [26], which was proposed based on the two-class SVM classifier. It is different from the univariate quadratic optimization problem in the case of the two-class classification using SVM classifier, and is aimed at solving the following multivariate quadratic optimal programming problems, when using the MC-SVM method to classify multiple types of targets:

(7) $$min(\frac{1}{2}\sum_{i=1}^{K}\sum_{j=1}^{i-1}{\left\|w_{i}-w_{j}\right\|}^{2}+\frac{1}{2}\sum_{i=1}^{K}{\left\|w_{i}\right\|}^{2})$$

subject to

(8) $${\left(w_{i}-w_{j}\right)}^{T}\cdot x_{i}-(b_{i}-b_{j})-1\geq 0\;i,j=1,2,\cdots ,K,i\neq j$$

where **w***_i_* and **w***_j_* are the parameters of the piecewise linear margin. *K* is the number of data categories to be classified, and the specific derivation process is available in the literature in Reference [26]. This method utilizes a piecewise nonlinear classification function constructed by a single quadratic program to classify a multi-class dataset. 

The MC-SVM method improved from principle, rapidly increases the algorithm complexity. For example, the computational complexity of the two-class SVM classifier is O(n), whereas that of the multi-class SVM classifier is O(n^M^), where M is the number of categories of targets to be classified. For WSN nodes with insufficient computing capacity, a multi-target classification algorithm based on multiple two-class classifiers is more appropriate. 

### 3.2. DAGSVM and Binary-Tree SVM

The more representative multi-classification algorithms based on two-class classifiers are DAGSVM and Binary-Tree SVM. These two methods use multiple two-class classifiers cooperatively, to classify multiple types of targets.

**Figure 3:**
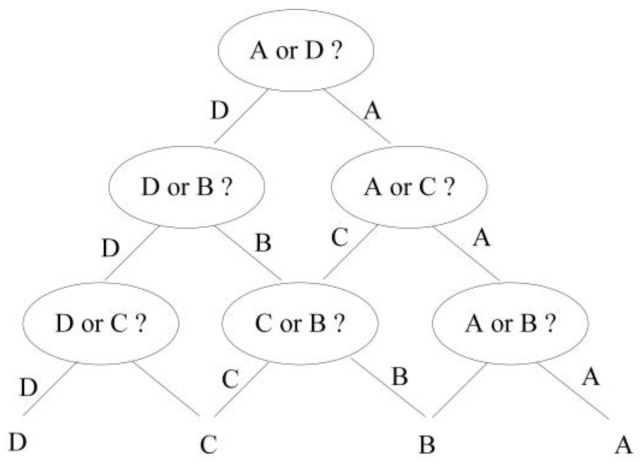
Architecture of directed acyclic graph SVM (DAGSVM) method.

The DAGSVM method constructs a directed acyclic graph that resembles a normal binary tree. The one-against-one classifier, trained at all the nodes shown in Figure 3, was used to classify the unclassified dataset, and finally determine the category of the dataset to be classified by cycle comparison.

Considering the multi-target classification of four types of targets as an example, and denoting the four types of targets as A, B, C, and D, the classification process of the DAGSVM algorithm is illustrated in Figure 3.

The number of one-against-one classifiers required by the DAGSVM multi-classification algorithm is $C_{N}^{2}=N\times (N-1)/2$. The parameter N is the number of target types. The classification accuracy of the DAG-SVM algorithm is significantly affected by the classification accuracy of the one-against-one classifier, at the key node in the directed acyclic graph. The classification accuracy of A is denoted as P*_ABCD_*(A), which is also the probability of accurately determining A from A, B, C, and D. Similarly, the classification accuracy of B, C, and D are denoted as P*_ABCD_*(A), P*_ABCD_*(B), and P*_ABCD_*(D), respectively. The formulas for calculating these values are as follows:

(9) $$P_{ABCD}\left(A\right)=P_{AD}(A)\cdot P_{AC}(A)\cdot P_{AB}(A)$$

(10) $$\begin{array}{ll} P_{ABCD}(B) & =P_{AD}\left(A|B\right)\cdot \left[P_{AC}\left(A|B\right)\cdot P_{AB}\left(B\right)+\left(1-P_{AC}\left(A|B\right)\right)\cdot P_{BC}\left(B\right)\right]\\ & +\left(1-P_{AD}\left(A|B\right)\right)\cdot P_{BD}\left(B\right)\cdot P_{BC}\left(B\right) \end{array}$$

(11) $$\begin{array}{ll} P_{ABCD}(C) & =P_{AD}\left(D|C\right)\cdot \left[P_{BD}\left(D|C\right)\cdot P_{CD}\left(C\right)+\left(1-P_{BD}\left(D|C\right)\right)\cdot P_{BC}\left(C\right)\right]\\ & +\left(1-P_{AD}\left(D|C\right)\right)P_{AC}\left(C\right)\cdot P_{BC}\left(C\right) \end{array}$$

(12) $$P_{ABCD}\left(D\right)=P_{AD}(D)\cdot P_{BD}(D)\cdot P_{CD}(D)$$

where P*_AD_*(A) is the classification accuracy of A in the one-against-one classifier (A/D), and P*_AC_*(A) is the classification accuracy of A in the one-against-one classifier (A/C). P*_AD_*(A|B) represents the probability that A is classified as B when using the one-against-one classifier (A,D), and P*_BD_*(D|C) represents the probability that C is classified as D when using the one-against-one classifier (B,D). The classification accuracy calculation Formulas (9)–(12), reveal that the target classification accuracy of the DAGSVM algorithm depends on the category number N, and the classification accuracy of the two-class classifiers at the node of the directed acyclic graph. Top-down error accumulation affects the classification performance of this method. For example, the classification accuracy of A (P*_ABCD_*(A)) is determined by the classification accuracy of three two-class classifiers (P*_AD_*(A), P*_AC_*(A), and P*_AB_*(A)), and the classification error of each two-class classifiers will reduce the final classification accuracy P*_ABCD_*(A). The most important factor affecting the classification accuracy of the two-class classifier is the geometric spacing between the training features of the two types of targets. The larger the geometric spacing is, the smaller the upper bound of the classification error rate is, i.e., the higher the classification accuracy that can be achieved by optimizing the parameter method.

### 3.3. Binary-Tree SVM

**Figure 4:**
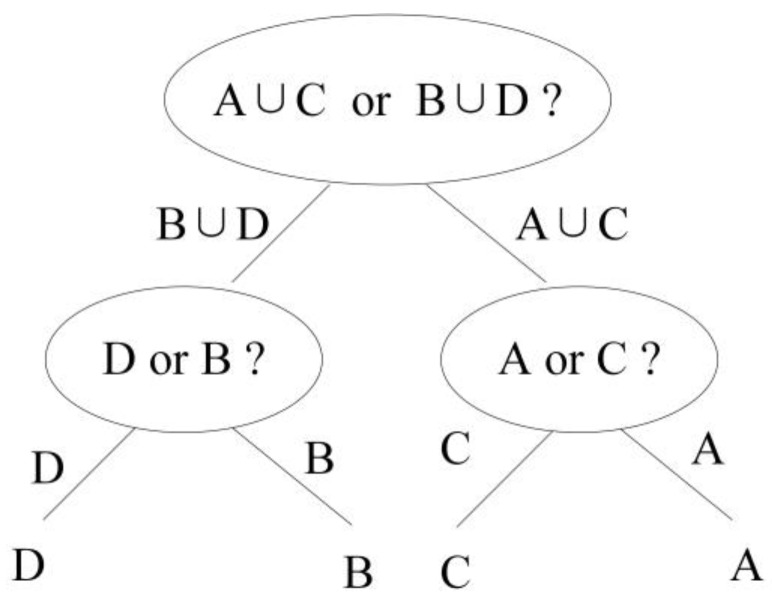
Architecture of binary-tree SVM method.

The binary-tree SVM classifier is also composed of multiple two-class SVM classifiers. Different from the DAGSVM method, the binary-tree SVM classifier first classifies the several types of targets into two major categories, and then continues to classify each of the major categories into two classes, until each class contains only one type of target. Considering the multi-target classification of four types of targets as an example, and denoting the four types of targets as A, B, C, and D, the classification process of binary-tree SVM is shown in Figure 4.

As shown in Figure 4, the first two-class classifier (A ∪ C/B ∪ D) is trained using the combined features, after the features of the targets A and C are combined to obtain the combination features (A ∪ C), and the features of B and D are combined to obtain the combination features (B ∪ D). The target features to be classified are first sent to the two-class classifier (A ∪ C/B ∪ D) for classification. After the classification result of the first classifier is obtained, assuming it to be (A ∪ C), the result is further sent to the two-class classifier (A/C) for the next level of classification.

The number of one-against-one classifiers required by the binary-tree SVM algorithm is (N − 1), where N is the number of target types. In this study, we classified four targets, and therefore, three two-class classifiers are required here. The calculation formulas for all the types of targets are as follows:

(13) $$P_{ABCD}\left(A\right)=P_{A\cup C,B\cup D}(A\cup C|A)\cdot P_{AC}(A)$$

(14) $$P_{ABCD}\left(B\right)=P_{A\cup C,B\cup D}(B\cup D|B)\cdot P_{BD}(B)$$

(15) $$P_{ABCD}\left(C\right)=P_{A\cup C,B\cup D}(A\cup C|C)\cdot P_{AC}(C)$$

(16) $$P_{ABCD}\left(D\right)=P_{A\cup C,B\cup D}(B\cup D|D)\cdot P_{BD}(D)$$

where P*_A∪C, B∪D_*(A ∪ C) represents the probability that target A or C is classified as A ∪ C, when using the two-class classifier (A ∪ C/B ∪ D). P*_AC_*(A) denotes the classification accuracy of target A, when using the two-class classifier (A/C).

The classification accuracy of the binary tree SVM method is as shown in Equations (13)–(16). As illustrated by the equations, combining multiple types of data into two major categories and sending it into the two-class SVM classifier significantly influences the final classification accuracy. If the classification result of the two-class SVM classifier (A ∪ C/B ∪ D) at the first layer is incorrect, it is not feasible to correct it in the subsequent classification process.

## 4. Voting Comparison Method

A total of N × (N − 1)/2 independent two-class classifiers are utilized to classify multiple targets, when using the DAG-SVM method. Compared to the other methods, the drawback of DAG-SVM is its excessive time consumption, both in the classifier-training step and in the step where trained classifiers are used to classify classification targets. The advantage of DAGSVM is its high classification accuracy, which results from the one-to-one comparison in the algorithm. The binary-tree SVM method uses N − 1 two-class classifiers to classify multiple targets and using fewer two-class classifiers, results in less time consumption. The classification accuracy of the binary-tree SVM algorithm is significantly affected by the classification accuracy of the classifier at the first layer. 

Combining the advantages of these two methods, we proposed a comparison voting method, named the cross-voting method.

**Figure 5:**
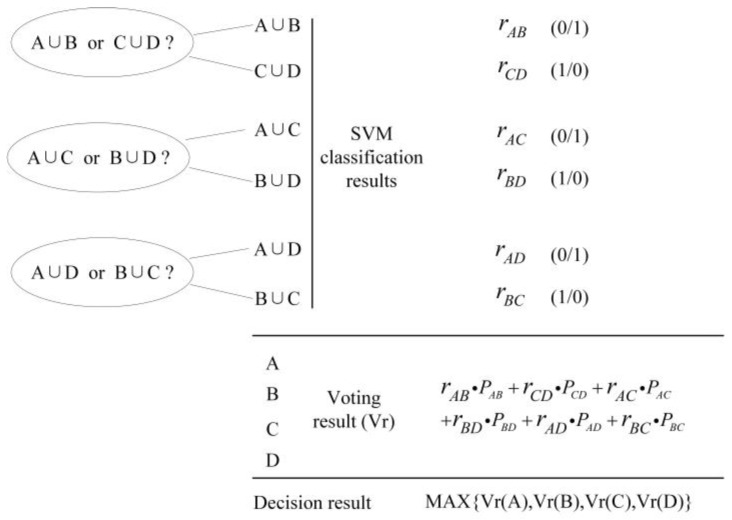
Flowchart of the cross-voting algorithm.

Figure 5 shows the flow of multi-target classification using the cross-voting algorithm, which can classify the signals of the four types of targets using three SVM classifiers. At first, the pairwise combination of the sample datasets of the four types of targets (A, B, C, and D) are treated as the new class dataset (A ∪ B, C ∪ D, A ∪ C, B ∪ D, A ∪ D, and B ∪ C), and these new datasets are utilized for training the three two-class classifiers (A ∪ B/C ∪ D, A ∪ C/B ∪ D, and A ∪ D/B ∪ C). 

The trained classifiers are subsequently used to classify the new data, and class voting is performed according to the classification result of each classifier. For example, if the classifier (A ∪ B/C ∪ D) classifies the new data as A ∪ B, the candidate results of classes A and B obtain a vote. Here, *r_AB_* and *r_CD_* are the results of the classifier (A ∪ B/C ∪ D). If the classification result of the new dataset is A ∪ B, *r_AB_* is equal to 1, and *r_CD_* is equal to 0. *P_AB_* is the voting weight. At the end of the algorithm, the votes of all the candidate results are counted, and the class with the highest ticket is the final classification result of the data to be classified.

If the voting weights of the results in the voting method are identical, there may be cases wherein the votes of several candidate classes are equal when the votes of all the candidates are counted.

The pairwise combinations of the sample datasets of the targets (A, B, C, and D) are treated as the new class dataset, and they train the three two-class classifiers (A ∪ B/C ∪ D, A ∪ C/B ∪ D, and A ∪ D/B ∪ C). It is assumed that the voting weights of *P_AB_* = *P_CD_* = *P_AC_* = *P_BD_* = *P_AD_* = *P_BC_* = 1. 

**Figure 6:**
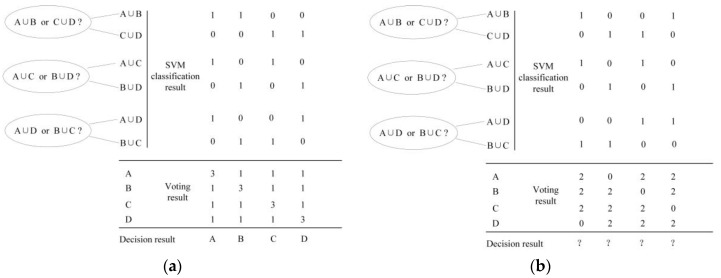
Two instances of multi-classification using the cross-voting algorithm: (**a**) in an ideal condition; (**b**) in the condition where it is unfeasible to classify.

The classification result of the target A should preferably resemble the first data column shown in Figure 6a. A vote is provided for classes A and B, according to the classification result (A ∪ B), a vote is provided for class A and category C, according to the classification result (A ∪ C) of the second classifier, and a vote is provided for classes A and D according to the classification result (A ∪ D) of the third classifier. After counting the votes of the respective candidate results of A, B, C, and D; the candidate class A obtains three votes, and the number of votes for the remaining classes (B, C, and D) is 1. Therefore, A is considered as the classification result.

In the process of using the cross-voting SVM classification algorithm to classify the target, there are likely to be cases wherein the votes of several candidate classes are identical when the distance between the different sample datasets is not large, that is, the difference between the target feature vectors is not large enough. In the cases shown in Figure 6b, the voting weights of the results in the voting method are identical, and the incorrect classification results of the two-class classifiers yields identical voting results for A, B, C, and D. In this case, the algorithm cannot determine the final classification result.

We add voting weights in the voting process to solve this problem. The higher the credibility of the classification result, the larger the weight assigned, and if the classification of the two-class classifier is lower, a smaller weight is assigned. After the weight is introduced, notwithstanding whether the classification result shown in Figure 6b appears, the classification result can still be obtained from the final (different) number of votes.

### 4.1. Class Distance and Classification Accuracy of Two-Class SVM Classifiers

When using the SVM classifier for target classification, the two-class classifier is first trained using the sample dataset. Then, the trained classifier is used to classify the dataset to be classified. Considering the target classification using the SVM method as an example, the seismic signal datasets of AAV3, DW3, AAV4, and DW4 are used as the training datasets, for training a two-class classifier. Then, the trained classifier is utilized to classify datasets of other runs. After the classifier is trained, the accuracy of the classification is determined mainly by the classifier and the data to be classified. The performance of the classifier is largely affected by the sample datasets used for training. We utilized the dataset introduced in Section 2.1, to study the performance of the two-class SVM classifier when using different training datasets. For studying the relationship between the class distance of the sample data used for training and the classification accuracy of the two-class classifier, we used different sample data to train the SVM classifier. The trained classifier was utilized to classify the remaining dataset of runs; and its average classification accuracy was then calculated.

The method for calculating the distance between the sample data utilized for training the two-class classifier is described in detail in Section 4.2.

**Figure 7:**
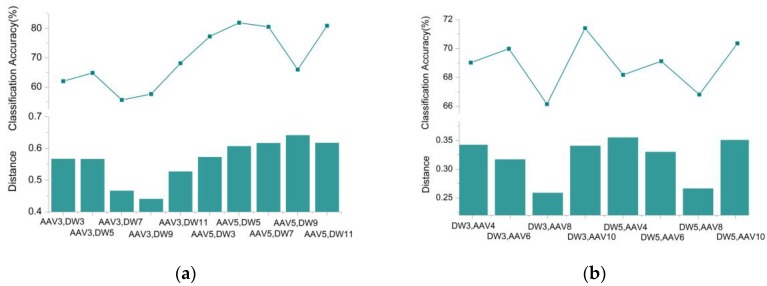
Relationship between SVM classification accuracy and distance between training datasets: (**a**) distance between AAV3/AAV5 and other DW datasets, and corresponding average classification accuracy; (**b**) distance between DW3/DW5 and other AAV datasets, and corresponding average classification accuracy.

Figure 7a shows the distance between the training datasets and the average classification accuracy of the other datasets of runs using the two-class SVM classifiers, when utilizing the following seismic signal datasets to train the SVM classifier: (AAV3, DW3); (AAV3, DW5); (AAV3, DW7); (AAV3, DW9), (AAV3, DW11); (AAV5, DW3); (AAV5, DW5); (AAV5, DW7); (AAV5, DW9); and (AAV5, DW11). The data used for training is the feature matrix of the target signal extracted using the WCER method, and the method described in Section 4.2 was used to calculate the distance between two feature matrices. Figure 7b shows the average classification accuracy of other datasets of runs using the two-class SVM classifiers, when utilizing the following seismic signal datasets to train the SVM classifier: (AAV4, DW3); (AAV6, DW3); (AAV8, DW3); (AAV10, DW3); and (AAV4, DW5); (AAV6, DW5); (AA8, DW5); (AAV10, DW5).

Figure 7 shows that the performance of the classifier is positively related to the distance of the sample dataset used when training the classifier. The larger the geometric distance, the higher the classification accuracy that can be achieved; the smaller the distance, the lower the classification accuracy of the classifier.

To highlight the credibility of the classification results when the distance of the training feature is large, we used different voting weights to adjust the classification results of different classifiers when using the voting method to integrate the results of different two-class SVM classifiers. When the distance of the training dataset of the classifier is large, the classification result has higher credibility, and the classification result is accorded a larger voting weight. Conversely, when the distance of the training dataset is small, the result of the classifier is accorded a small voting weight.

### 4.2. Distance of Training Dataset and Voting Weight

The acquired vehicle signals generated from the detected vehicle targets in WSN. The features of the acoustic signals of these four types of targets were then extracted using the WCER feature extraction method. The feature matrixes of the acoustic signals of the four targets are represented by A, B, C, and D.

(17) $$A=\begin{array}{c} \begin{array}{cccc} w_{a1} & w_{a2} & \cdots & w_{aM} \end{array}\\ \left|\begin{array}{cccc} a_{11} & a_{21} & \cdots & a_{M1}\\ a_{12} & a_{22} & \cdots & a_{M2}\\ \vdots & \vdots & \vdots & \vdots \\ a_{1N} & a_{2N} & \cdots & a_{MN} \end{array}\right| \end{array}\;B=\begin{array}{c} \begin{array}{cccc} w_{b1} & w_{b2} & \cdots & w_{bM} \end{array}\\ \left|\begin{array}{cccc} b_{11} & b_{21} & \cdots & b_{M1}\\ b_{12} & b_{22} & \cdots & b_{M2}\\ \vdots & \vdots & \vdots & \vdots \\ b_{1N} & b_{2N} & \cdots & b_{MN} \end{array}\right| \end{array}$$

As illustrated by Equation (17), **A** and **B**, which are N × M matrices, represent the feature matrices of the acoustic signals of two targets. The number of rows in the matrix N, denotes the number of segments N, that the target signal corresponding to A was divided into. When using the WCER method to extract features from a signal sequence, segmentation is performed every 1024 points to extract the features. M is the number of variables of each line in the matrix, which is also twice the wavelet decomposition depth number in the WCER feature extraction method. The parameter w*_ai_* represents the *i*th eigenvector of the feature matrix **A**, and w*_bi_* represents the *i*th eigenvector of the feature matrix **B**. The different columns in the matrix react to the characteristics of the target signal in different dimensions. Therefore, when calculating the distance between two feature matrices, the distance between the feature variables in the same column of the two feature matrices is first calculated using Equation (18).

(18) $$d(w_{ai},w_{bi})=\sqrt{(w_{ai}-w_{bi}){\left(w_{ai}-w_{bi}\right)}^{T}}=\sqrt{\sum_{j=1}^{N}{\left(a_{ij}-b_{ij}\right)}^{2}}$$

where *a_ij_* is the *j*th variable in the *i*th eigenvector of the feature matrix **A,** and *b_ij_* is the *j*th variable in the *i*th eigenvector of the feature matrix **B**. There are *M* eigenvectors (**w**_1_, …, **w***_M_*) in the feature matrix of the target signal. After calculating the distance between the eigenvectors of the feature matrix, it is also necessary to quantify the distance between the feature matrices, which is the distance between the target signals. The distance between the matrices is calculated by the formula below:

(19) $$d(A,B)=\frac{1}{M}\sum_{i=1}^{M}d(w_{ai},w_{bi})=\frac{1}{M}\sum_{i=1}^{M}\sqrt{\sum_{j=1}^{N}{\left(a_{ij}-b_{ij}\right)}^{2}}$$

In the cross-voting method, the merged sample datasets of the multiple targets are utilized to train the two-class SVM classifier. Therefore, based on the distance between the feature matrices of a single target, it is necessary to further calculate the distance between the merged datasets of the multiple targets. We used the Lance–Williams dissimilarity update formula [27], to calculate the distance between the single target dataset A and the union dataset of multiple targets (B ∪ C):

(20) $$d(A,(B\cup C))=\alpha_{B,C}d(A,B)+\alpha_{C,B}d(A,C)+\beta d(B,C)+\gamma \left|d(A,B)-d(A,C)\right|$$

using the following equation to set the parameters:

(21) $$\alpha_{B,C}=\frac{\left|N_{B}\right|}{\left|N_{B}\right|+\left|N_{C}\right|},\alpha_{C,B}=\frac{\left|N_{C}\right|}{\left|N_{B}\right|+\left|N_{C}\right|},\beta =0,\gamma =0$$

where *N_B_* is the number of rows in feature matrix **B**, and *N_C_* is the number of rows in the feature matrix **C**, which is also the number of segments of the target signal corresponding to the feature matrix **C**. Using Equation (20) for further derivation, we can obtain the distance between the union dataset (A ∪ D) and the merged dataset (B ∪ C):

(22) $$\begin{array}{l} d((A\cup D),(B\cup C))=\alpha_{A,D}d(A,(B\cup C))+\alpha_{D,A}d(D,(B\cup C))\\ =\alpha_{A,D}(\alpha_{B,C}d(A,B)+\alpha_{C,B}d(A,C))+\alpha_{D,A}(\alpha_{B,C}d(B,D)+\alpha_{C,B}d(C,D)) \end{array}$$

where α**_A_**_,**D**_ and α**_D_**_,**A**_ are the adjustment coefficients and can be calculated by Equation (21).

After calculating the distance between the union dataset and the union dataset yielded by using Equation (22), the distance between the aggregation datasets is mapped to the voting weight range of [0.7, 1.3]; this voting weight range is selected to ensure that the sum of two classifier result weights are larger than the largest classifier result weight. The distance between the aggregation datasets in the three SVM classifiers in the voting method are denoted as *Dist*(*1*), *Dist*(*2*), and *Dist*(*3*). We used Equation (23) to calculate the voting weight of the result of each classifier.

(23) $$P(i)=\frac{2}{\sqrt{2\pi }}e^{-2NorD^{2}(i)}+0.5$$

(24) $$NorD(i)=\frac{Dist(i)-Max(Dist(i))}{Max(Dist(i))-Min(Dist(i))}+\sigma$$

where *NorD*(*i*) is the normalized distance between the aggregation datasets, and *P*(*i*) is the voting weight of the result of the *i*th SVM classifier. To ensure that the value of the voting weight *P*(*i*) is within the range of [0.7, 1.3], then |*NorD*(*i*)|, which is the absolute value of *Dist*(*i*) after normalization, should have a value range of [0.2, 0.8]. Since the value range of $\frac{Dist\left(i\right)-Max\left(Dist\left(i\right)\right)}{Max\left(Dist\left(i\right)\right)-Min\left(Dist\left(i\right)\right)}$ is [−1, 0], to make the value of |*NorD*(*i*)| satisfy the condition, the preferable range of the adjustment coefficient *δ* is [0.2, 0.8], and here we set *δ* to 0.3.

### 4.3. Average Segmentation of Training Data

**Figure 8:**
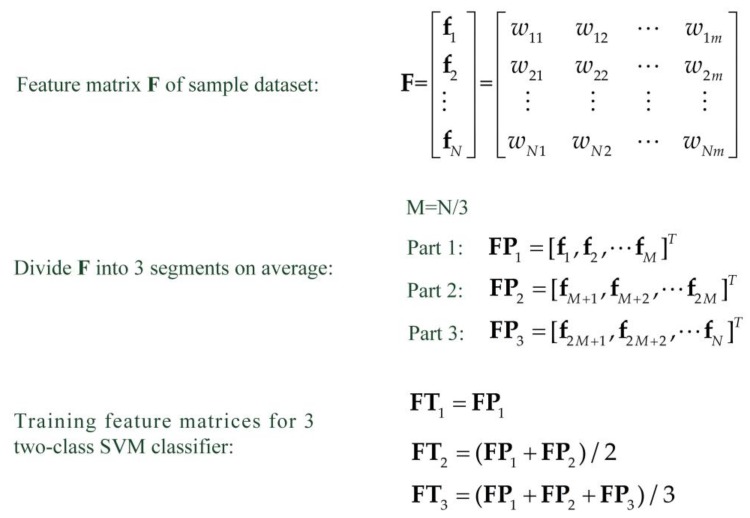
Average segmentation of the training feature matrix.

The algorithm classifies the new input features using the pre-trained two-class SVM classifiers. The classification result is then voted out. Partial training datasets with large differences from the mean values of the sample data, are likely to cause incorrect assessments when using the trained SVM classifier to classify the input new features, which is likely to result in low performance of the cross-voting algorithm. Additionally, using the same training dataset to train the three two-class SVM classifiers cannot maximize the performance of the result comparison of the different classifiers in the algorithm, which is the main characteristic of the algorithm. Therefore, the segmentation averaging method shown in Figure 8 is utilized to process the training dataset. Through this approach, the training datasets of the three SVM classifiers maintain a certain degree of difference, and the influence of the wild points on the classification performance of the classifier in the training data are eliminated as much as possible.

### 4.4. Cross-Voting SVM Classification Algorithm

The algorithm flow of the cross-voting comparison method when there are four types of targets is illustrated in Algorithm 1. When the number of targets is five or six (in general, for WSN application system, the number of vehicle types to be classified does not exceed six), it is only necessary to change the aggregation datasets in the two-class SVM classifiers.

**Algorithm 1** Cross-Voting Algorithm for 4 Targets Classification   Input: Training dataset: Feature matrices {AFT, BFT, CFT, DFT} of 4 target signals (A, B, C, D). Feature vector X of unknown target.  Output: Vehicle type of feature vector X.  (1) Using the method shown in Figure 8 to segmentally average the training data to obtain a different target signal features matrix for training classifier. {(AFT1, AFT2, AFT3); (BFT1, BFT2, BFT3); (CFT1, CFT2, CFT3); (DFT1, DFT2, DFT3);}  (2) Using the obtained feature matrix to construct different training datasets and train three classifiers separately:  using ((AFT1 ∪ BFT1), (CFT1 ∪ DFT1)) to train the first two-class classifier 1;  using ((AFT2 ∪ CFT2), (BFT2 ∪ DFT2)) to train the second two-class classifier 2;   using ((AFT3 ∪ DFT3), (BFT3 ∪ CFT3)) to train the third two-class classifier 3;  (3) Calculating the distance between the training datasets for the three classifiers using Equation (22):  Dist (1): D((AFT1 ∪ BFT1), (CFT1 ∪ DFT1));   Dist (2): D((AFT2 ∪ CFT2), (BFT2 ∪ DFT2));   Dist (3): D((AFT3 ∪ DFT3), (BFT3 ∪ CFT3));   Calculating the voting weight of the three classifier results P(i) (i = 1, 2, 3), using Equations (23) and (24);  (4) Classifying the feature vector X using the trained three classifiers, i.e., classifier 1, classifier 2, and classifier 3, and obtain the classification result: (rAB, rCD); (rAC, rBD); (rAD, rBC);  (5) Counting vote results of 4 different targets using the classification results of classifier 1, classifier 2, and classifier 3.  Vr(A) = rAB·P(1) + rAC·P(2) + rAD·P(3);  Vr(B) = rAB·P(1) + rBD·P(2)+ rBC·P(3);  Vr(C) = rCD·P(1) + rAC·P(2) + rBC·P(3);  Vr(D) = rCD·P(1)+ rBD·P(2) + rAD·P(3);  (6) Finding Max{Vr(A), Vr(B), Vr(C), Vr(D)} and the corresponding vehicle type is the vehicle type of feature vector X;

## 5. Performance of Cross-Voting SVM Algorithm

The datasets provided by the SensIT experiment consisted of acoustic signals, seismic signals, and infrared signals for two types of vehicles (AAV and DW). The infrared signals of the AAV vehicle and DW vehicle were pulse signals, and the frequency distribution of the signals was almost the same. The features of the infrared signals extracted by the WCER method had no recognizability, which made them unable to be utilized as different class signals. To study the performance of the cross-voting SVM method for multi-vehicle classification, we utilized the acoustic and seismic signals of AAV to represent two vehicle signals, and the acoustic and seismic signals of DW were utilized to represent two other vehicle signals.

### 5.1. Classification Accuracy of Cross-Voting SVM Algorithm

The features extracted from runs AAV3, AAV4, DW3, and DW4 were utilized for training the SVM classifier. Subsequently, the trained classifier was used to classify the target using the features extracted from the dataset of the other 15 runs (AAV5–AAV11 and DW5–DW12). The Radial Basis Function (RBF) function was used as the kernel function of the SVM classifier, and the gamma value in the kernel function was set to 0.5.

Table 2 illustrates that the three multi-target classification methods based on the two-class SVM classifier can effectively classify multiple vehicles detected in the WSN. For revealing the performance of these three methods more clearly, we calculated the overall average classification accuracy and time consumption of the vehicle classification using these methods. The overall average classification accuracy was calculated based on the average classification accuracy of a single target class. The classification accuracy of the *i*th type of target was denoted as *Aca(i)* (*i* = 1, 2, …, *C*), and the overall average classification accuracy, *Oaca*, is

(25) $$Oaca=\frac{1}{P}\overset{P}{\underset{i=1}{\sum }}Aca\left(i\right)$$

where *P* is the number of target categories for all the targets that have been classified. Here, there were four vehicles categories.

Table 3 presents the overall average classification accuracy and average time consumption of the various multi-class classification methods based on the SVM classifier. The results demonstrated that the average classification accuracy using the cross-voting SVM method was the highest, and that the time consumption of the method was satisfactory. The time required for the DAGSVM method was the highest of the three, and the average classification accuracy of this method was lower than that of the cross-voting SVM. Compared to the cross-voting method and DAGSVM method, the binary-tree SVM method exhibited the lowest classification accuracy and the lowest time consumption. In summary, the cross-voting SVM method significantly improved the classification accuracy of multi-target classification, in terms of time consumption. Compared with the DAGSVM method and binary-tree SVM method, the classification accuracy improved by approximately 7% when the cross-voting SVM method was used.

The main advantage of the cross-voting SVM method was the increased acoustic signal classification accuracy of DW7–DW12, compared to other methods. The main reason for the low acoustic signal classification accuracy of DAGSVM and Binary-tree SVM for DW7–DW12, was that the acquired acoustic signals were very similar to the acoustic signal generated by AAV, and a single two-class SVM classifier is easy to misclassify. Since the DAGSVM and binary-tree SVM methods used the same training set to train each classifier, there was no diversity of training samples and no further cross-validation steps after classification, resulting in low classification accuracy when classifying the acoustic signals of DW7–DW12. The classification accuracy of acoustic signals of DW9, obtained by these three methods (the DAGSVM method, binary-tree SVM method, and cross-voting SVM method) were quite different, so we utilized the acoustic signal of DW9 as an example to compare the differences in the classification processes of the three methods.

**Figure 9:**
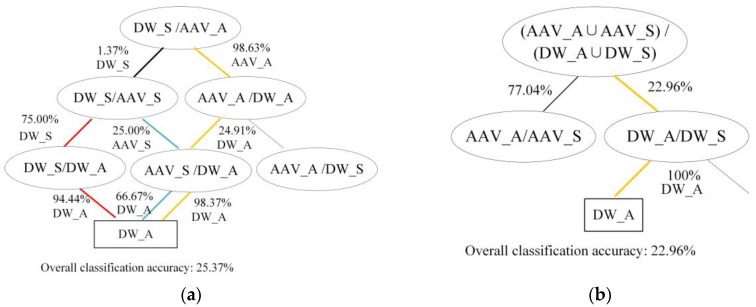
Classification accuracy of each step when using DAGSVM method: (**a**) and binary-tree SVM method; (**b**) to classify acoustic signal of DW9.

Figure 9a,b indicate the classification accuracy for the acoustic signal of DW9 at each step, which is also the classification accuracy of each two-class classifier, when using the DAGSVM method and binary-tree SVM method, respectively. In the DAGSVM classification process, the probability that the classifier (DW_S/AAV_A) classified the acoustic signal of DW9 as non-DW_S was 98.63%. However, the classification accuracy of the next layer classifier (AAV_A/DW_A) was only 24.91%, which made the overall classification accuracy unsatisfied. In the binary-tree SVM classification process, the first-level classifier (AAV_A ∪ AAV_S/DW_A ∪ DW_S) had a probability that the acoustic signal of DW9 was classified to be the correct class (DW_A ∪ DW_S) of 22.96%. The low classification accuracy of the first-level classifier, determined that the overall classification accuracy would not exceed 22.96%, even though the classification accuracy of the next level classifier reached 100%.

Table 4 shows that the classification accuracy of each step when using the cross-voting SVM method classified the acoustic signal of DW9. The cross-voting method utilized multiple classifiers and verified the classification results of these classifiers, to reduce the impact of low classification accuracy of a single classifier on the overall classification accuracy. In the cross-voting SVM classification process, the classification accuracy of the classifier (AAV_A ∪ DW_S/AAV_S ∪ DW_A) in the three classifiers was only 19.75%, but the classification accuracies of the other two classifiers, (AAV_A ∪ DW_A/AAV_S ∪ DW_S) and AAV_A ∪ DW_S/AAV_S ∪ DW_A) were 73.71% and 98.16%, respectively. The overall classification accuracy rate reached 72.21% after the voting link of the algorithm, which effectively prevented the influence of the third classifier’s bad classification performance on the overall classification results.

### 5.2. Cross-Voting Method Using Different Two-Class Classifier

In addition to the SVM method, other commonly used two-class classifiers include the NB, TreeBag method, adaboosting method, and KNN method. The two-class SVM classifier in the cross-voting SVM algorithm can be replaced with these two-class classifiers. We replaced the two-class SVM classifier in the cross-voting SVM algorithm with the NB, TreeBag, and adaboosting classifiers. The vehicle classification results are presented in Table 4.

The average classification accuracy and average time consumption of the cross-voting method, when using the different two-class classifiers are presented in Table 5. The results demonstrated that the cross-voting method using the DT two-class classifier, adaboosting two-class classifier, or SVM classifier could effectively classify the targets in the WSN system. When using the TreeBag classifier as the basis two-class classifier in the cross-voting method to classify the vehicles in the WSN, the classification accuracy reached approximately 76%; however, the time consumption was excessive. Compared with the TreeBag two-class classifier, the time consumption decreased significantly when using the NB classifier as the basis two-class classifier in the cross-voting method, and the classification accuracy of multi-target classification simultaneously decreased by approximately half. The average classification accuracy obtained by using the SVM classifier as the basis two-class classifier in the cross-voting method was 1% lower than that obtained using the NB classifier, but the time consumption decreased by 90% when using the SVM two-class classifier. Compared to using the SVM classifier as the basis two-class classifier in the cross-voting method, the average classification accuracy obtained using the adaboosting classifier was lower, whereas the time consumption was higher. In summary, the SVM two-class classifier is the most suitable classifier amongst the studied classifiers and can be utilized in the cross-voting method for multi-target classification in a WSN system.

### 5.3. Comparison with Other Multi-Class Classification Method

In addition to the multi-class classification algorithm based on the two-class classifier, there are certain methods originally capable of multiple class classifications. Among these methods, the NB method and adaboosting method are widely used. We utilized these three methods to classify the four vehicle signals mentioned above, and the results were compared with the classification results of the cross-voting method.

As shown in Table 6, the average classification accuracy obtained using the cross-voting SVM method is the highest among the three methods, being approximately 10.7% higher than that obtained using the NB method when classifying the multiple vehicles in a WSN system. This is mainly because the prior probability model obtained by analyzing the training dataset in the NB method is inaccurate, when the sample dataset is not sufficient. The classification accuracy obtained using the adaboosting method to classify the multiple targets was the lowest among the three methods.

We additionally observed that the time consumed by the proposed cross-voting SVM method to classify the vehicles detected by a WSN system was less than that consumed by the adaboosting method. Moreover, the time consumption of the NB method was significantly less than that of the other three methods.

Deep learning methods based on multilayer perception and neural networks, are state-of-the-art algorithms for multi-class classification. To classify the four vehicle signals mentioned above, we utilized a feedforward neural network (FFNN) [28], a deep belief network (DBN) [29], which was an alternative class of deep neural network (DNN), and the extreme learning machines (ELM) [30] algorithm, and the results were compared to the classification results of the cross-voting method. In addition to classification accuracy and average time consumption of classification, the training time before classification and the memory consumption of the algorithms were also recorded for comparison. Both the DBN method and FFNN methods utilize a dual hidden layer network to perform classification; the two hidden layers of the DBN network contain 300 hidden units and 100 hidden units, and the two hidden layers of the FFN network contain 300 hidden units and 200 hidden units. The number of neurons in the ELM network that we utilized was 300, and the sigmoid function was utilized in the hidden neurons of ELM.

As shown in Table 7, the average classification accuracy obtained using the DBN method and FFNN are higher than that obtained using the cross-voting SVM method, when classifying the multiple vehicles in a WSN system, and classification accuracy may be higher after optimizing the DBN method and FFNN network. The classification accuracy obtained when using the ELM method was the lowest among these methods, being approximately 2.6% lower than that obtained using the cross-voting SVM method. The results in Table 7 also indicate that, although the average classification accuracy obtained using the ELM method and cross-voting SVM method is lower than that obtained using the DBN and FFNN method, their training time and memory consumption are more satisfactory than those obtained using the DBN and FFNN method. Excessive memory requirements (9253.94 MB for DBN and 34,660.9 MB for FFNN), made these two methods impractical for vehicle classification in WSN systems with limited computing capacity and memory resources. Using a neural network-based approach on a computer may be a sensible choice when offline classifying a large amount of multiple vehicle signals, acquired by multiple sensor nodes in the WSN system. Although the training time and classification time consumption of the cross-voting SVM method were slightly higher than those of the ELM method, the higher accuracy and lower memory requirements made the cross-voting SVM method more suitable than the ELM method, for multiple vehicles classification in a WSN system. 

## 6. Conclusions

In this paper, we proposed a novel multi-class target classification method, named the cross-voting SVM method, based on the SVM classifier for vehicle classification in WSNs. The SVM classifier was selected as the basis two-class classifier for vehicle classification, after comparing the classification performances of several commonly-used classification methods. We then combined the advantages of the two available methods, the DAGSVM method and the binary-tree SVM method, for multi-class target classification based on the SVM classifier. We subsequently proposed the cross-voting SVM method. Finally, we studied the target classification accuracy and time consumption of the proposed target classification method. The experimental results demonstrated that the classification accuracy of the targets detected in the WSN, was effectively improved by using the cross-voting method to classify vehicle targets in the WSN system.

## Figures and Tables

**Table 1:** Average classification accuracy and time required for different classification methods using acoustic features. KNN—k-nearest neighbor; DT—decision tree; NB—naïve Bayes; AdaBoost—adaptive boosting; SVM—support vector machine.

	KNN	DT	NB	AdaBoost	SVM
Average classification accuracy	72.22%	52.82%	65.71%	69.83%	71.03%
Average time required for classification	1.4783 s	0.0024 s	0.0102 s	0.0743 s	0.1440 s

**Table 2:** Classification accuracy of four types of target signals using the cross-voting SVM method, DAG SVM method, and binary-tree SVM method.

Run Name	Cross-Voting SVM		DAG SVM		Binary-Tree SVM	
	A	S	A	S	A	S
AAV5	99.77%	41.14%	99.32%	44.08%	99.66%	41.02%
AAV6	98.44%	47.92%	93.07%	49.74%	96.45%	40.92%
AAV7	93.67%	45.01%	89.90%	46.56%	91.24%	42.86%
AAV8	93.50%	42.37%	92.34%	48.67%	93.67%	43.34%
AAV9	97.64%	45.80%	99.78%	51.32%	100%	46.24%
AAV10	82.33%	54.17%	90.77%	54.79%	94.68%	52.23%
AAV11	89.75%	38.10%	90.03%	42.04%	90.31%	36.03%
Average	93.59%	44.93%	93.60%	48.17%	95.14%	43.23%
DW5	68.58%	86.13%	80.97%	82.00%	73.40%	88.40%
DW6	86.31%	89.93%	85.94%	91.68%	84.26%	96.58%
DW7	73.71%	88.40%	25.37%	86.37%	22.96%	90.70%
DW8	69.81%	75.72%	31.02%	69.65%	28.16%	76.22%
DW9	72.21%	74.88%	7.26%	73.10%	5.82%	76.70%
DW10	67.17%	92.38%	33.69%	90.97%	29.24%	95.43%
DW11	56.28%	98.52%	47.11%	96%	45.00%	99.21%
DW12	61.53%	91.36%	40.96%	87.19%	39.65%	94.11%
Average	70.58%	87.16%	44.03%	84.62%	41.06%	89.67%

**Table 3:** Comparison of average classification accuracy and time consumption using the cross-voting SVM method, DAG SVM method, and binary-tree SVM method.

	Cross-Voting SVM	DAGSVM	Binary-Tree SVM
Overall average classification accuracy	74.52%	67.39%	67.15%
Time Consumption of Classification	0.3255 s	1.1207 s	0.2796 s

**Table 4:** Classification accuracy of each step when using the cross-voting SVM method to classify the acoustic signal of DW9.

	Classification Accuracy	Overall Classification Accuracy
Classifier 1 (AAV_A ∪ AAV_S/DW_A ∪ DW_S)	73.71% (DW_A ∪ DW_S)	72.21% (DW_A)
Classifier 2 (AAV_A ∪ DW_A/AAV_S ∪ DW_S)	98.16% (AAV_A ∪ DW_A)	
Classifier 3 (AAV_A ∪ DW_S/AAV_S ∪ DW_A)	19.75% (AAV_S ∪ DW_A)	

**Table 5:** Average classification accuracy and time consumption of the cross-voting method using different two-class classifier.

	Cross-Voting NB	Cross-Voting DT	Cross-Voting Adaboosting	Cross-Voting SVM
Overall average classification accuracy	37.48%	75.64%	72.55%	74.52%
Time Consumption of Classification	0.0253 s	4.1748 s	1.0750 s	0.3255 s

**Table 6:** Comparison of average classification accuracy and time consumption using different multi-class classification algorithms.

	NB	Adaboosting	Cross-Voting SVM
Overall average classification accuracy	63.85%	53.52%	74.52%
Time Consumption of Classification	0.0077 s	0.3621 s	0.3255 s

**Table 7:** Comparison of average classification accuracy, time consumption, and memory consumption using different multi-class classification algorithms.

	DBN	FFNN	ELM	Cross-Voting SVM
Overall average classification accuracy	78.40%	75.76%	71.88%	74.52%
Time Consumption of Training	350.84 s	452.36 s	0.5581 s	15.1698 s
Average Time Consumption of Classification	0.0298 s	0.0452 s	0.1783 s	0.3255 s
Memory Consumption	9253.94 M	34,660.9 M	369.02 M	123.95 M
